# Nanobiopsy investigation of the subcellular mtDNA heteroplasmy in human tissues

**DOI:** 10.1038/s41598-024-64455-0

**Published:** 2024-06-14

**Authors:** Alexander Bury, Angela Pyle, Amy E. Vincent, Paolo Actis, Gavin Hudson

**Affiliations:** 1 Wellcome Centre for Mitochondrial Research, Biosciences Institute, Faculty of Medical Sciences, Newcastle University, Newcastle, UK; 2 Wellcome Centre for Mitochondrial Research, Translational and Clinical Research Institute, Faculty of Medical Sciences, Newcastle University, Newcastle, UK; 3 NIHR Biomedical Research Centre, Faculty of Medical Science, Newcastle University, Newcastle, UK; 4 School of Electronic and Electrical Engineering and Pollard Institute, University of Leeds, Leeds, UK; 5 Bragg Centre for Materials Research, Leeds, UK

**Keywords:** Molecular biology, Electrical and electronic engineering, Nanobiotechnology, Genomics

## Abstract

Mitochondrial function is critical to continued cellular vitality and is an important contributor to a growing number of human diseases. Mitochondrial dysfunction is typically heterogeneous, mediated through the clonal expansion of mitochondrial DNA (mtDNA) variants in a subset of cells in a given tissue. To date, our understanding of the dynamics of clonal expansion of mtDNA variants has been technically limited to the single cell-level. Here, we report the use of nanobiopsy for subcellular sampling from human tissues, combined with next-generation sequencing to assess subcellular mtDNA mutation load in human tissue from mitochondrial disease patients. The ability to map mitochondrial mutation loads within individual cells of diseased tissue samples will further our understanding of mitochondrial genetic diseases.

## Introduction

Inherited and somatic mitochondrial DNA (mtDNA) variation is a significant contributor to human disease^1^. Inherited mtDNA variation is associated with a wide range of diseases that can present from birth to old age^2–4^. In parallel, somatic mtDNA mutations have been shown to accumulate with age and preferentially accumulate in specific organs or tissues, and typically lead to late-onset disease such as Parkinson’s disease (PD)^5–7^, Alzheimer’s disease (AD)^8^, and pathological ageing^5,9,10^. Organelle, specifically mitochondrial, heterogeneity at the single-cell level is associated with localised physiological function^11,12^ and can be characteristic of aberrant cellular pathways in disease^4,13^. Previous work has suggested that low-level heteroplasmic variation is a common occurrence at the single cell level^14^ and that the clonal expansion of these low-level variants affects mitochondrial function^14,15^ and contributes to disease^16–18^. However, the mechanism of clonal expansion appears to vary between mtDNA point mutations and mtDNA deletions and depending on the tissue in question^19–21^. Some of these mechanisms, such as the clonal expansion of mtDNA point mutations in mitotic tissues, are better understood^10,22^. Conversely, the mechanisms driving clonal expansion of mtDNA variants in post-mitotic tissues remain elusive^22,23^. The tissue and mutation specific nature of both mtDNA heteroplasmy and clonal expansion means that, to better understand these disease mechanisms, it is necessary to study mtDNA mutation within a well characterised tissue^24,25^.

In addition to mtDNA sequence characteristics^26^, work in early developmental tissues suggests that factors such as mitochondrial locality, particularly proximity to the nucleus, influence heteroplasmy dynamics^27,28^. Further studies investigating heteroplasmic variation have shown that variation can be regional, and it has been hypothesised that subcellular environment and cellular structure can impact clonal expansion^29,30^. Whilst stochastic models have been used to describe the mode of clonal expansion^30–33^, they require estimates of mutation rates specific to the tissue and mutation in question and are based on limited spatial information^18,34^.

Previous studies have been able to assess the spatially restricted expansion of mtDNA mutations longitudinally in skeletal muscle fibres and sarcomeres or, to a limited extent, able to investigate traverse patterns of mtDNA mutations across muscle fibres^29,30^. However, longitudinal analysis alone cannot take into account the clonal expansion of mtDNA mutations within a branched, three-dimensional mitochondrial network and mitochondrial subpopulations can exist in foci smaller than the permitted sampling range of existing technologies^29,35^. Skeletal muscle tissue is a well characterised sample medium in the investigation of clonal expansion of mtDNA mutations^29,32,36^, which makes it a strong candidate for our studies.

The advent of nanoprobe technologies^37,38^ presents a unique opportunity to study mtDNA heteroplasmy with a sufficient sampling resolution, simultaneously preserving the spatial information needed to study somatic mtDNA variation and clonal expansion. A range of micro- and nanoprobe-based technologies have been recently developed to enable the localized probing of cells and tissues^39–43^. This includes the use of the nanobiopsy setup to longitudinally profile the transcriptome of cancer cells^44^. Our group has also recently developed a subcellular nanobiopsy method based on scanning ion-conductance microscopy (SICM) to enable the subcellular sampling of mitochondria from human tissues with a spatial resolution superior to the gold standard technique, laser capture microdissection (LCM)^45^. Nanobiopsy is a form of scanning probe microscopy comprising a glass micro- or nanopipette controlled by a nanomanipulator, which can manoeuvre the pipette to subcellular regions, facilitated by fluorescence microscopy to achieve highly precise sampling of subcellular material^39,46^. Nanobiopsy has been successfully used to study mtDNA obtained from mitochondria isolated from cultured fibroblasts^39^ and human skeletal muscle tissue^46^ and some of the recent applications of this technology have been recently reviewed^48^.

**Figure 1:**
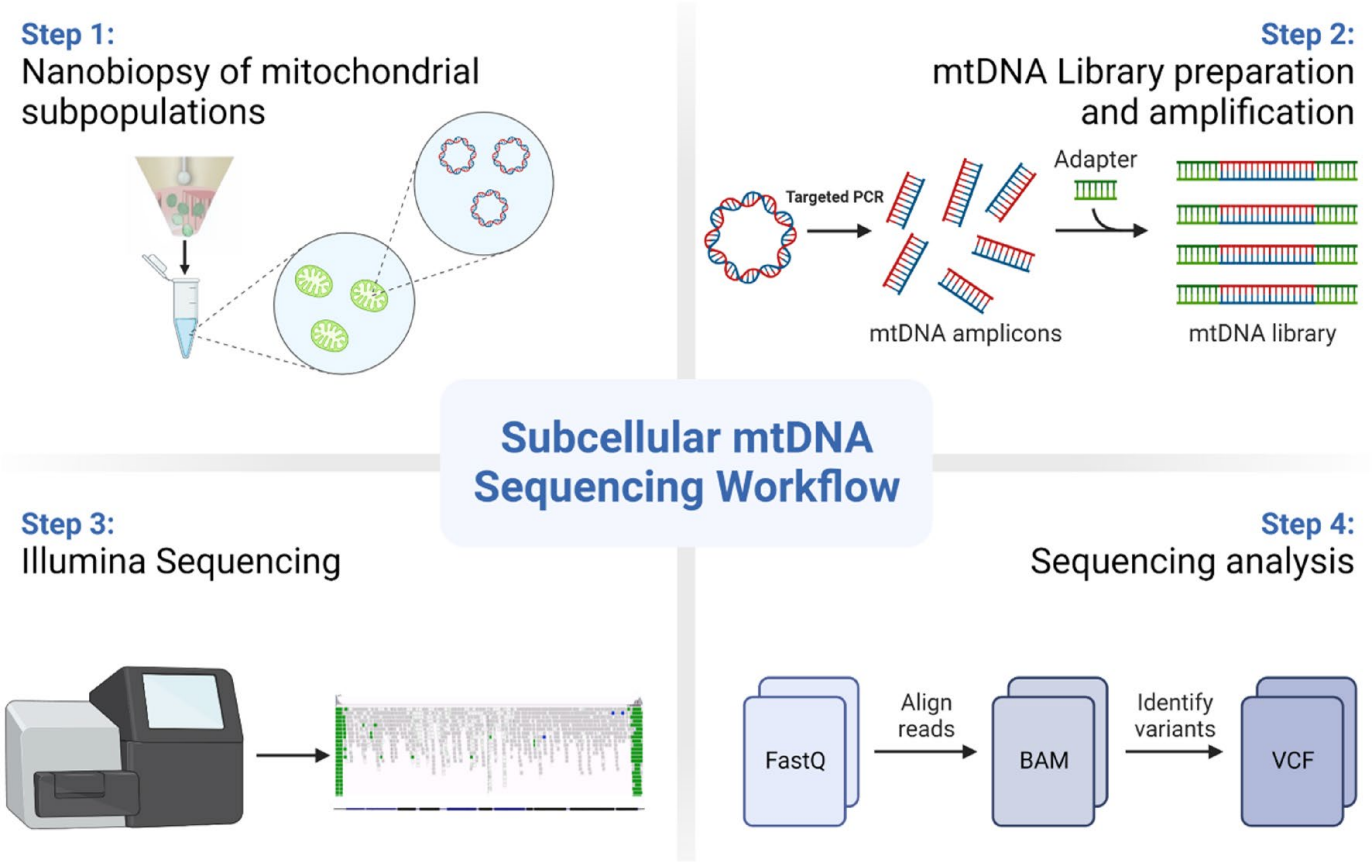
A graphic showing the workflow for the sequencing of mtDNA isolated from subcellular mitochondrial populations obtained using nanobiopsy. Following the nanobiopsy of fluorescently labelled mitochondria from distinct subcellular foci, mitochondria are lysed and mtDNA molecules are fragmented prior to library preparation and clean-up. MiSeq sequencing was performed on the control region of mtDNA and post sequencing analysis allowed alignment of reads and identification of mtDNA variants. In Step 3 the integrated genomic viewer image is adapted from Wei and colleagues^49^.

In this study, we demonstrate that nanobiopsy is a viable tool for the study of heteroplasmic mtDNA variants in human tissue. This proof of concept work shows that nanobiopsy can be used in conjunction with next-generation sequencing to investigate sub-cellular mtDNA heterogeneity (Fig. 1).

## Materials and methods

### Skeletal muscle tissue biopsies

Excess skeletal muscle tissue from hamstring was collected during anterior cruciate ligament surgery with prior informed consent and ethical approval from the Newcastle Research Ethics Committee (REC: 12/NE/0267). Tissue used in this study was from a single healthy control (Female, 23 years). Tissue was requested from the biobank and approved (REC: 16/NE/0267—Application Ref: MRBOC ID039) and all work is performed with ethical approval from the Newcastle and North Tyneside Local Research Ethics Committee. All methods using human tissue were performed in accordance with the relevant guidelines and regulations outlined by the Newcastle and North Tyneside Local Research Ethics Committee, the University of Leeds and Newcastle University. All research was carried out with proper HTA licensing and tissue transfer agreements in place.

### Cryosectioning

Skeletal muscle tissue was cryosectioned into multiple 15 µm sections. Sections were mounted on square glass microscope slides (Agar Scientific Ltd). Sections were then air-dried for 1 h and stored at − 80 °C prior to staining.

### Immunofluorescent staining

Immunofluorescent staining was performed on skeletal muscle tissue sections fixed with 4% PFA, using primary antibodies raised against mitochondrial proteins: VDAC1, NDUFB8 and MT-CO1 (Table 1). Dehydration and rehydration of tissue sections, through a methanol gradient, was performed to permeabilise sections. Blocking and immunofluorescent staining steps were performed as previously described, with the addition of DAPI staining to allow visualisation of myonuclei^50^.

**Table 1:** Quad-immunofluorescence primary and secondary antibodies.

Target	1^o^ antibody	Product code	Supplier	Dilution	2^o^ antibody	Product code	Supplier	Dilution
Myonuclei	DAPI	D9542	Sigma-Aldrich	1:400	–	–	–	–
NDUFB8	Mouse anti-NDUFB8 (IgG1)	ab110242	Abcam	1:100	Biotin Anti-Mouse IgG1;	S32357	Life technologies	1:200
					Strepavidin Alexa Fluor 647	S21374	Life technologies	1:100
MT-CO1	Mouse anti-MT-CO1 (IgG2a)	ab14705	Abcam	1:100	Alexa Fluor 433 Anti-Mouse IgG2a	S21131	Life technologies	1:200
VDAC1	Mouse anti-VDAC1 (IgG2b)	ab14734	Abcam	1:100	Alexa Fluor 546 Anti-Mouse IgG2b	A21143	Life technologies	1:200

All primary and secondary antibodies are listed, used for QIF staining of specific targets in skeletal muscle tissue. The product number, supplier, dilution and antibody target are also included.

### Sub-cellular nanobiopsy

**Figure 2:**
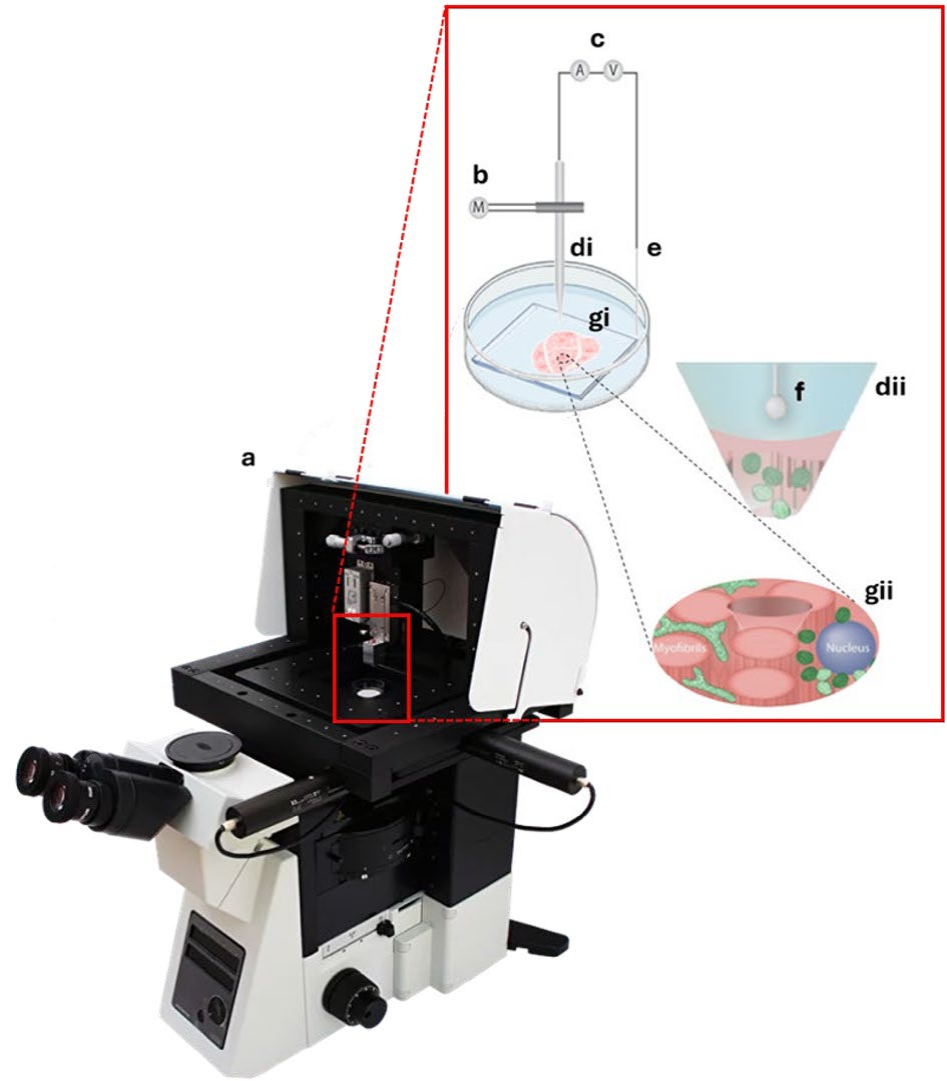
The nanobiopsy platform can be integrated into an inverted fluorescent or confocal microscope set-up. (**a**) The nanobiospy apparatus is housed within a Faraday cage to minimise electromagnetic noise (**a**). The nanobiopsy apparatus itself comprises a piezo motor which facilitates sub-micron manipulation of the micropipette (**b**), an amplifier (**c**) which modulates the current and voltage within the working (**f**) and reference electrodes (**e**) and a pipette (**d**). Control of the nanobiopsy is achieved and co-ordinated using the Scanning Ion-Conductance Microscope controller and corresponding software. The sample of interest is immersed in an aqueous solution and as the pipette moves towards the sample, the ion current passing between the working (**f**) and reference (**e**) electrodes is increasingly occluded allowing real-time feedback of relative pipette position. This facilitates the highly-precise entry into and sampling from specimens such as human skeletal muscle tissue (**g**). Adapted from Bury et al., 2022^45^.

Nanobiopsy was performed as previously described^39,45^. Micropipettes were fabricated from borosilicate glass capillaries (BF100-50-7.5, Sutter Instruments), using an SU-P2000 laser puller (World Precision Instruments). Nanobiopsy was performed using an SICM comprising an Axon MultiClamp 700B amplifier (Molecular Devices), MM-6 micropositioner (Sutter Instruments), p-753 linear actuator (Physik Instrumente), pE-300 LED illumination system (CoolLED) and an Eclipse Ti2 inverted microscope (Nikon) (Fig. 2)^45^. Control of the micropipette and electrochemical measurements were executed using the SICM (ICAPPIC Ltd.)^46^ and Axon pClamp 11 (Molecular Devices) software^46,47^. The automated approach of skeletal muscle fibres was achieved with a fall distance of 2 µm, fall rate of 100 µm/s and a 1% current set-point. Nanobiopsies were obtained from three different foci within skeletal muscle fibres: perinuclear (PN), subsarcolemmal (SS) and intermyofibrillar (IMF).

### mtDNA isolation, enrichment, and next-generation sequencing

Briefly, the human mtDNA control region (m.1-573 and m.16024-16569) was enriched as overlapping PCR amplicons using a high fidelity TaKaRa PrimeSTAR GXL DNA polymerase and four primer sets, described previously (TaKaRa; Table 2^51^). A hot start protocol was followed (40 cycles): 98 °C for 10 s, 60 °C for 30 s, 68 °C for 1 min, then held at 4 °C. Successful PCR was assessed using gel electrophoresis on a 1% agarose gel. PCR products were then pooled in equimolar concentrations, and purified using Agencourt AMPure XP beads (Beckman-Coulter, USA). Next-generation sequencing (NGS) was used to sequence subcellular and single-cellular lysate samples, as previously described^6,52^. Briefly, pooled amplicons underwent tagmentation, amplification, Ampure-bead purification and were normalized using an Illumina Nextera XT DNA sample preparation kit (Illumina, CA, USA). Multiplex pools were sequenced using MiSeq Reagent Kit v3.0 (Illumina, CA, USA) in paired-end, 250 bp reads. Postrun data as FASTQs, limited to reads with QV ≥ 30, were exported for analysis. NGS of nanobiopsied mitochondria was compared to lysate obtained from single-cells collected by LCM^29,53^ and control human genomic DNA (gDNA) (G304A, Promega), sequenced using BigDye terminator sequencing (Applied Biosystems) and previoulsy reported methods^16^.

**Table 2:** Targeted PCR primers.

Amplicon	PCR product size (bp)	Forward primer sequence	Forward primer position	Reverse primer sequence	Reverse primer position
D1	830	5'-ATCGGAGGACAACCAGTAAG-3’	m.15758–15777	5'-AGGGTGATAGACCTGTGATC -3’	m.1–19
D2	475	5'-CTCAACTATCACACATCAACTG-3’	m.16223–16244	5'-AGATACTGCGACATAGGGTG -3’	m.110–129
D3	410	5'-CCTTAAATAAGACATCACGATG-3’	m.16548–16569	5'-CTGGTTAGGCTGGTGTTAGG -3’	m.370–389
D4	448	5'-GCCACAGCACTTAAACACATC-3’	m.323–343	5'-TGCTGCGTGCTTGATGCTTG -3’	m.752–771

PCR product size and primer sequences (and positions) for both forward and reverse primers. D1-D4 correspond to amplicons spanning the entirety of the mtDNA control region. Primer positions are in reference to the revised Cambridge reference sequence (NC_012920.1).

### Bioinformatics analysis

Informatics analysis of FASTQ files was performed as previously described^54^. Raw reads were assessed and filtered using FASTQC (v0.11.7; Babraham Bioinformatics, Cambridge, UK), aligned to the human reference genome (hg38) using BWA (v0.7.15; Li and Durbin, 2009) and sorted and indexed using Samtools (v1.3.1). Duplicate reads are marked and removed using Picard (v2.2.4). Variant calling was performed using VarScan (v.2.4.3) with the following parameters: ≥ 1000× coverage; >  > 1000×; support reads, > 10 ≥ base quality, > 30; > mapping quality, > 10; > variant threshold, < 0.05. Variant annotation was performed using VEP (v109).

### Statistical analysis and graphics

Data was analysed using Prism v5 using data appropriate statistical tests (detailed in the text). Statistical significance was set at p < 0.05. Figures were produced using Prism v5 and BioRender (BioRender.com).

## Results

### Nanobiopsy

**Figure 3:**
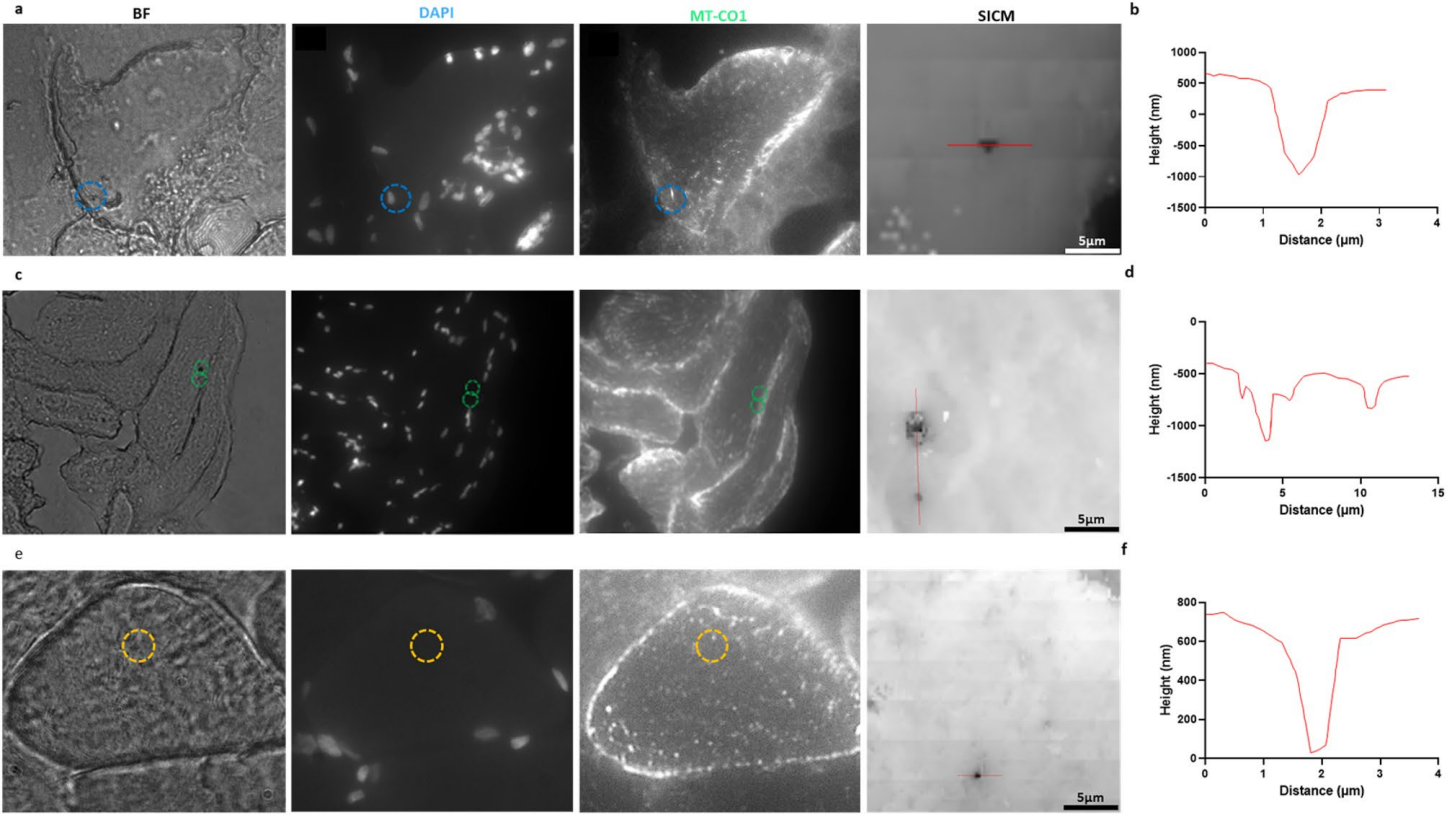
Post-biopsy images were taken from one of three distinct subcellular foci from human skeletal muscle fibres: perinuclear (PN, blue, top); subsarcolemmal (SS, green, middle) or intermyofibrillar (IMF, yellow, bottom). The PN nanobiopsy corresponds to biopsy lysate sample 22 (fibre 4), the SS to biopsy lysate samples 25 and 26 (fibre 5) and the IMF to biopsy lysate sample 51 (fibre 6). Post-biopsy images were taken corresponding to bright-field (BF), DAPI nuclear stain and mitochondrial respiratory chain Complex IV, subunit I (MT-CO1; **a, c, e**). SICM topographical images (**a, c, e**) and scans (**b, d, f**) were also taken to evidence the successful acquisition of nanobiopsies.

**Figure 4:**
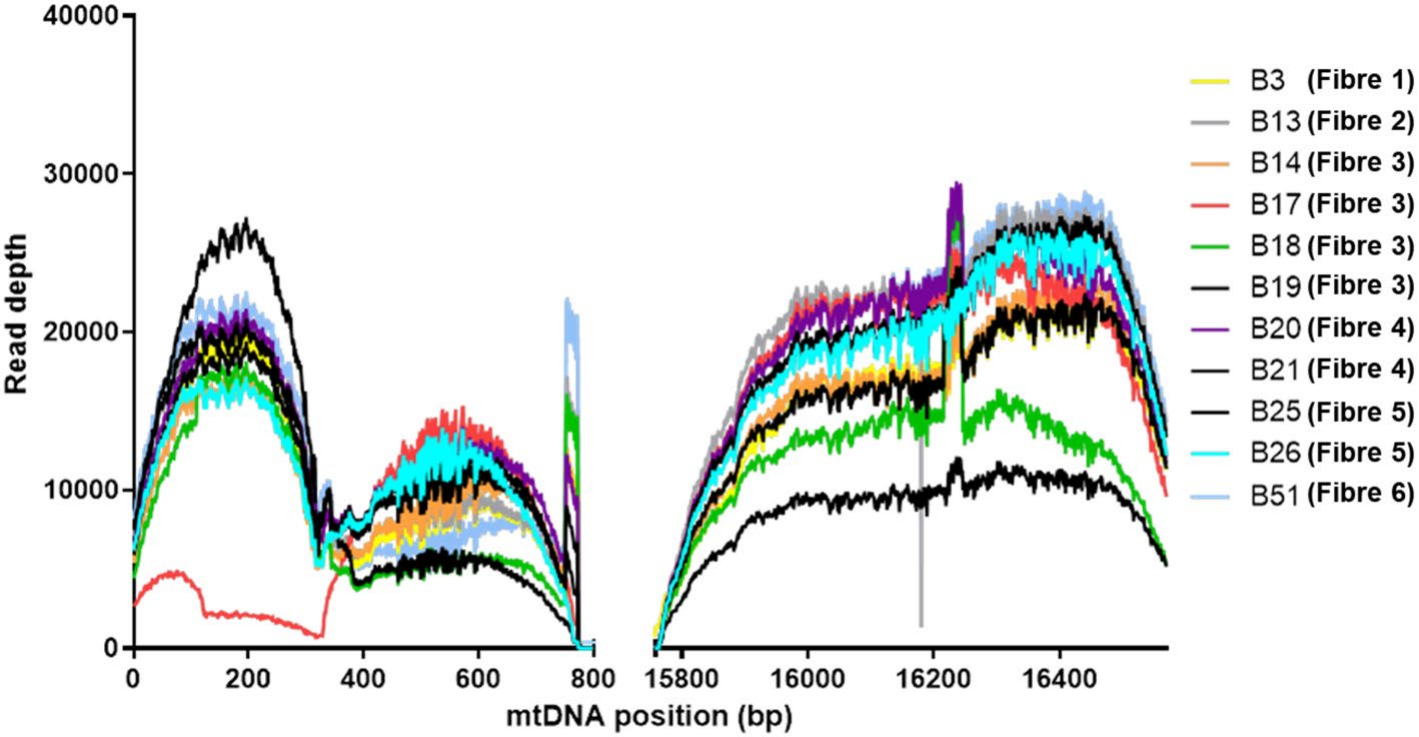
A plot showing the breadth of coverage of Illumina MiSeq spanning the entirety of the mtDNA control region. Each plotted line corresponds to the read depth of mtDNA sequenced from individual nanobiopsy lysate samples acquired from human skeletal muscle tissue. The depth of sequencing coverage ranges from 0 to 29430. The mean depth of coverage was 10911-fold. Peaks in the read depth correspond to D2 and D4 primer binding sites at mt.750 and mt.16250 (Table 2).

MtDNA from four (of twenty-three) single-cell and eleven (of fifty) nanobiopsy lysate samples (Fig. 3, Table S1), were isolated from human skeletal muscle tissue (> 1 ng input) and successfully sequenced. All samples, both nanobiopsies and single-cells, were taken from tissue obtained from a single healthy control (*n* = 1). Samples were successfully enriched using targeted PCR and then sequenced. Nanobiopsy lysate underwent NGS (MiSeq, Illumina) with an average sequencing depth of > 10,000 (Fig. 4). This is in line with previous cell-culture based nanobiopsy experiments^39^ and other subcellular isolation techniques^41,42^ and compares favourably with traditional single-cell approaches such as LCM^45,55–57^. Single-cell lysate samples were Sanger sequenced alongside two positive gDNA controls.

### Subcellular mtDNA sequencing

**Figure 5:**
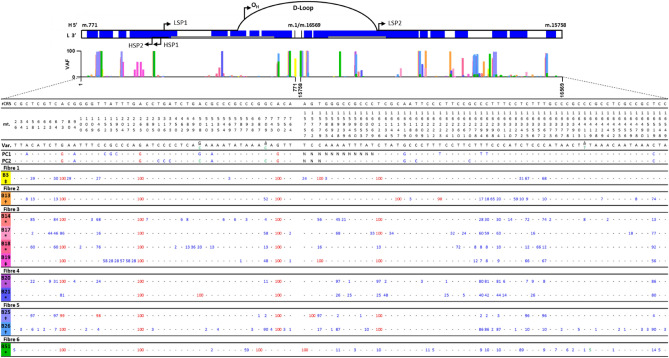
The top graphic represents all sequenced nanobiopsy variants (blue) mapped to the control region of the mitochondrial genome (black). Grey bars correspond to hypervariable segments (HVS) of the mitochondrial genome: HVS1 (left) and HVS2 (right). Also depicted are the heavy strand replication origin (O_H_), as well as heavy (HSP) and light stand promoters (LSP)^58^. The bar graph shows the mitochondrial variant allele frequencies (VAF, %) corresponding to colour-coded nanobiopsy lysate samples. Positive control (PC) gDNA samples were sequenced using Sanger sequencing and are represented by the substituted bases—heteroplasmic (blue) or homoplasmic (red). Nanobiopsies (B*n*) underwent MiSeq sequencing (Illumina) and are listed by fibre and nanobiopsy lysate number—heteroplasmic (blue) or homoplasmic (red). The positions of all mitochondrial single nucleotide variants (mtSNVs) are noted relative to the rCRS (NC_012920.1). For nanobiopsy lysate samples, numbers correspond to VAF. Subcellular foci of nanobiopsies are noted as: double dagger : PN; dagger: SS; asterisk: IMF; dash: wild-type. *N*  not successfully sequenced.

The eleven successfully sequenced nanobiospy lysate samples were taken from 6 individual skeletal muscle fibres (Fig. 5; Table S1): fibre 1: biopsy 3; fibre 2: biopsy 13; fibre 3: biopsies 14, 17–19; fibre 4: biopsies 20 and 21; fibre 5: biopsies 25 and 26 (Fig. 3c, d) and fibre 6: biopsy 51 (Fig. 3e, f). Where multiple biopsies were taken from the same fibre, they were all taken from the same serial section and all biopsies were taken from tissue obtained from a single healthy control (*n* = 1). Biopsies were also taken from one of three distinct foci within skeletal muscle fibres: PN, SS and IMF. The corresponding foci of each biopsy is labelled in Fig. 5, Table S1. Following NGS, 12 homoplasmic variants were detected in all nanobiopsy lysate samples compared with three homoplasmic variants in control gDNA: mt.263A > G was observed in all nanobiopsy and gDNA samples, mt.750A > G was observed in all gDNA and ten nanobiopsy lysate samples, mt.73A > G was observed in all gDNA and nine nanobiopsy lysate samples and mt.16129 T > C was observed in six nanobiopsy lysate samples but neither gDNA controls (Fig. 5). Heteroplasmic mtSNVs (% VAF in brackets) were also observed, such as mt.73A > G in fibres 3 (86%) and 4 (81%), whilst mt.16126 T > C was observed at heteroplasmic levels in fibre three: biopsy 18 (13%), fibre four: biopsies 20 (97%) and 21 (25%) and absent from fibre two.

The mt.750A > G mtSNV was not observed in fibre 6 and mt.16126 T > C was observed at only 10% (VAF; Fig. 5). This supports the clonal expansion or loss of mutations at the subcellular level, in disparate fashion to the tissue consensus.

A total of 103 mtDNA single nucleotide variants (mtSNVs) were detected in the control region of nanobiopsy lysate samples. This was a significantly greater number than gDNA control region variants (p < 0.01, Student’s t-test; Fig. 5). An average of 20 unique mtSNVs per biopsy were sequenced, which is comparable with previous studies^59^. This is compared with a sum of 21 unique mtSNVs in positive control gDNA. Of the 103 distinct mtSNVs from nanobiopsy lysate samples, 86 (39%) were low-level heteroplasmy (< 10%)^11^. Some heteroplasmies were consistent across the majority of cells e.g., mt.16519 in biopsy 13 (74%), fibre two; biopsies 17 (77%) and 18 (92%), fibre three; biopsies 20 (86%) and 21 (80%), fibre four and biopsy 26 (90%), fibre five. Some heteroplasmic variants were specific to individual cells e.g., mt.80C > A (29%) in fibre 1 and in fibre 4 mt.16134C > T (biopsy 20: 2%, biopsy 21: 48%). Other specific variants, e.g., mt.709 and mt.16294 in fibre 3, showed evidence of expansion between and within cells, with heteroplasmy levels ranging between 4–58% and 7–60% respectively (Fig. 5). This mtSNV is also present in biopsy 13 (fibre 2, VAF: 52%), biopsy 20 (fibre 4, VAF: 4%) and in biopsies 25 and 26 (fibre 5, VAFs: 2% and 90%). A number of biopsies from other fibres do not harbour this mtSNV. Collectively this shows that sequencing of samples acquired using nanobiospy, facilitates determination of heteroplasmy at the inter and intracellular level. Notably, we observed no nDNA variant detection in any biopsy.

**Figure 6:**
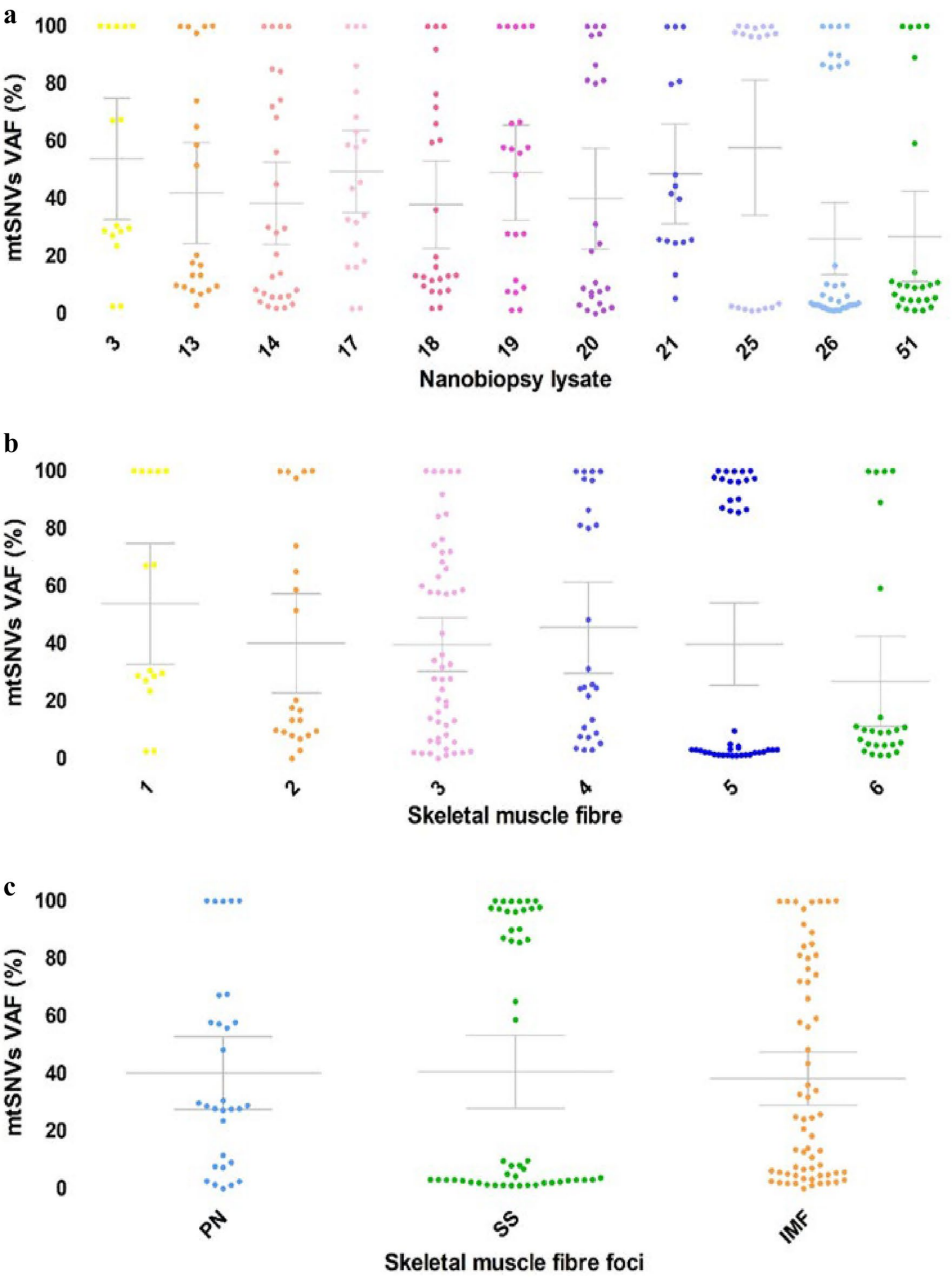
Data points represent the percentage Variable Allele Frequency (VAF) of the individual mitochondrial single nucleotide variants (mtSNVs). The error bars depict the mean allele frequency and 95% CI. (**a**) There was a significant difference in median heteroplasmy between individual nanobiopsy lysate samples (p = 0.004, Kruskal–Wallis test). (**b**) Although a difference in heteroplasmy between nanobiopsy samples was observed, this did not reach statistical significance (p = 0.054, Kruskal–Wallis test). (**c**) Biopsies were taken from three subcellular foci: Perinuclear (PN; *n* = 2, mean = 40.2, 95% CI = 27.6–52.9; mean median = 28.8), subsarcolemmal (SS; *n* = 3, mean = 40.6, 95% CI = 27.9–53.3; mean median = 7.5) and intermyofibrillar (IMF; *n* = 6, mean = 38.3, 95% CI = 29.1–47.5; mean median = 24.4). No significant difference in heteroplasmy between the different foci was observed (p = 0.555, Kruskal–Wallis test).

There was a significant difference in total heteroplasmy level between all nanobiopsy lysate samples (p < 0.01, Kruskal–Wallis test; Fig. 6a) but only moderate significance in heteroplasmy between individual fibres (p = 0.05, Kruskal–Wallis test; Fig. 6b) and no significant difference between skeletal muscle fibre foci (p > 0.05, Kruskal–Wallis test; Fig. 6c). Some biopsies exhibited a wide range of heteroplasmy (biopsies 3–21; Fig. 5a), whilst others showed a more bi-modal distribution of heteroplasmy (biopsies 25,26,52; Fig. 6a). This was also reflected in skeletal muscle fibres, with fibres one to four showing a greater spread of heteroplasmy and a bi-modal heteroplasmy distribution being observed in fibres five and six (Fig. 6b).

## Discussion

Single-cell level analysis has been fundamental to advancements in the field of mitochondrial biology, enabling the understanding of how mtDNA heterogeneity contributes to ageing^10,60,61^, neurodegenerative disease^5,8,62^ and cancer^63–65^—specifically through the proposed clonal expansion of mtDNA point mutations in germ line cells^10,18,32^ and of mtDNA deletions in post-mitotic cells^29,36,66^. However, we are fast approaching the point where single-cell analysis is no longer sufficient to characterise the function of mtDNA heterogeneity at the subcellular level^12,21,29^. In this study we demonstrate, for the first time, the subcellular sequencing of mtDNA acquired from subcellular skeletal muscle foci using nanobiopsy in tandem with  NGS.

A long-standing challenge in analysing low-level heteroplasmy has been the ability of sequencing technologies to accurately distinguish low-level signals from noise^67–69^. Although the advent of NGS technologies has made this possible^14^, remaining challenges include the inherent noise associated with bulk population analysis when trying to distinguish single- or subcellular level heteroplasmy. Subcellular sequencing, within neurons, has been demonstrated as being capable of detecting down to ~ 3% heteroplasmy^59^. Here we take this to the next level, in terms of sampling precision and sequencing resolution. There was a significantly greater number of successfully sequenced heteroplasmic nanobiopsy variants compared with gDNA variants. This could partly be attributed to the greater sequencing depth and efficiency of the MiSeq platform^70^. We do accept that being unable to sequence gDNA and single-cell lysate samples using the MiSeq format is a limition of this study—especially since it is more difficult to accurately sequence variants with a heteroplasmy level < 20% when using Sanger sequencing^70^. Despite this, the number of sequenced gDNA and single-cell mtSNVs is comparable (~ 5 to 25 mtSNVs) with previous bulk or single-cell studies adopting Sanger or Illumina sequencing^70–74^. The number of subcellular mtSNVs is notably greater even when compared with other studies investigating subcellular heteroplasmy^59^. The higher base substitution rate observed within the hypervariable segments of the control region could partly explain this higher number of individual variants^23,75^. Indeed, the majority of variants reported by Morris and colleagues occurred within the control region^59^. Subcellular sequencing of the entire mitochondrial genome is also likely to reveal a proportionately lower number of mtSNVs. Every effort was made to avoid the inclusion of contaminants, PCR bias and artefacts that could impact on the accuracy of reported heteroplasmy. Amplification of subcellular mtDNA concentrations, approaching the single molecule level, does help avoid many potential sources of PCR bias including NUMTs and PCR artefacts but are also more susceptible to co-amplifcation of contaminants^76,77^. A number of measures were employed to avoid PCR artefacts and bias, including use of sterile apparatus within a UV hood and use of a high fidelity polymerase^76^. A further potential refinement could be implemented by reducing the number of PCR cycles but this could come at the cost of being unable to amplify small subcellular quantities of mtDNA^78^. Even employing these controls there is a possibility that PCR artefacts could have impacted the reported levels of heteroplamsy and the only way to validate the results reported in this study is by repeating these assays with a greater sample size and ideally sequencing the entire mitochondrial genome for context. The observed elevation in nanobiopsy heteroplasmy is likely to be indicative of subtle subcellular ‘microheteroplasmy’ that was previously indistinguishable from the background noise associated with the sequencing of single-cells or homogenate^24,79^.

As with previous studies employing single-cell sequencing of the D-loop, there was an asbence of low level heteroplasmy corresponding to key elements within the control region, such as the LSP, likely due to the essential and conserved role that these regions play in maintaining mitochondrial health through mtDNA maintenance and this contrasts with the increase in heteroplasmy observed within the hypervariable segments^73^. Whilst very low-level nanobiopsy heteroplasmy (< 1%) was detected with MiSeq, 1% heteroplasmy was set as a threshold for analysis. Whilst not the focus of this study, investigating the origins (inherited or stochastic), role and possible clonal expansion of very low level heteroplasmy would be helpful in further understanding how pathogenic mutations are able to exceed threshold levels^32^. Indeed, it has been proposed that very low heteroplasmy (≤ 1%) in the D-loop is less subject to negative selection pressures and therefore able to be inherited and could clonally expand from birth^14,74^, alongside clonal expansion of stochastic mutations that may occur in early life^10,18^.

We were able to successfully enrich four out of twenty-three single-cell lysate samples and eleven out of fifty nanobiopsy lysate samples samples, a success rate of approximately 1:5. This is consistent with enrichment of very low concentrations of mtDNA using single molecule PCR^76,80^. The success rate of mtDNA enrichment is reduced further when enriching larger amplicons. PCR enrichment of the entire mitochondrial genome at single molcule concentrations is technically challenging and beyond the remit of this proof of principle study and therefore enrichment of the control region for this proof of principle serves as a well characterised alternative^76,81^, as adopted in other studies investigating subcellular mtDNA heteroplasmy^59,82,83^. However, for further studies, enrichment of the entire mitochondrial genome would be necessary to assess all mtDNA heteroplasmy and to do this we would propose multiple amplicons and possibly multiple round of PCR^71,80^.

Homoplasmic variants (≥ 98%) observed in gDNA were also observed in nanobiopsy samples and correspond to haplogroup markers^68,84^, though not all sequenced homoplasmic mtSNVs were observed in gDNA samples. This was likely to have been affected by the partial sequencing of positive control D1 amplicons, which coincides with the hypervariable segment 1 (HSV1)^73^. Compared to previously published studies, it is worth noting that some homoplamsic mtDNA variants were common between nanobiopsied samples and single-cell or bulk cell samples, including: mt.73A > G, mt.263A > G, mt.750A > G, 15779 T > C, 16126 T > C which correspond to haplotype markers^70,73,85,86^. Some homoplasmic mtSNVs were exclusive to certain fibres (e.g. mt.16147C > T, Fibre 1), whilst other mtSNVs were observed at varying heteroplasmic levels between and within skeletal muscle fibres, at positions corresponding to well characterised homoplasmic variants (< 98%;^70,73,85,86^). This could indicate the loss of mtDNA variants over time, consistent with previous studies investigating random genetic drift^14,32^. Alternatively, this may reflect ‘hierarchical heteroplasmy’: just as a tissue may be be heteroplasmic overall but can contain homoplasmic cells and vice versa, it is equally concievable that heteroplasmy level may differ between all mitochondria collectively within a whole cell and different mitochondrial subpopulations^87^. Until now, such patterns could not be investigated without a means to be able to precisely sample mitochondria from different subcellular foci. This would need to be verified in future assays but does perhaps indicate that some patterns of heteroplasmy and homoplasmy are being masked by cellular noise at the level of single-cell analysis. It was not possible to obtain data on variant allele frequency from Sanger sequenced samples and this limited the direct comparison of nanobiopsied samples with single-cell and gDNA samples. We also recongise that in place of a mixed ‘gDNA’ sample, mtDNA obtained from tissue homogenate (ideally from the same tissue biopsy as single-cell and nanobiopsies were taken) would be more representative in allowing us to determine the differences in heteroplasmy at the tissue, single-cell and subcellular level. This is something that we endeavour to incorporate into our methodology to investigate subcellular heteroplasmy in disease and control samples, in addition to other proposed refinements such as increasing the sample size and sequencing the whole mitochondrial genome of test samples.

The observed difference in heteroplasmy between nanobiopsies is reflective of the genetic heterogeneity that exists not just between cells but within subcellular mitochondrial populations. Whilst the difference in overall heteroplasmy load, between different fibres, did not reach significance, the specific polymorphisms and range of heteroplasmy that contribute to total heteroplasmy are different between different fibres. No significant difference was observed in heteroplasmy level between different subcellular foci, however, this could be affected by lack of complete sets of biopsies, where all foci were sampled within the same fibre, for direct comparison. The observed differences in heteroplasmy distribution between different nanobiopsies, foci, and fibres, whilst exciting, cannot yet be fully explained. Different patterns of heteroplasmy could reflect different mutation thresholds^15,23^. This may also be reflective of stochastic or selective clonal expansion events^21^, which has already been demonstrated in the change in heteroplasmy between mother and offspring of maternally inherited mtSNVs^73^.

In the context of other subcellular isolation technologies, coupled with either Sanger or Illumina seqencing, nanobiopsy performed objectively better in terms of number of mtSNVs within the control region that were successfully sequenced compared with single-cell micromanipulation-based formats, which were able to successfully sequence 6 (2—human, 4—mouse^59^) and 31 control region mtSNVs respectively^82^. This is comparable with other single-cell studies and previous demonstrations of the use of nanobiospy to isolate and sequence mtDNA from cultured fibroblasts^39^. Fluid Force Microscopy, incorporating atomic force microscopy, is similarly capable of highly precise subcellular mitochodrial isolation. Single molecule real-time sequencing (PacBio) was used to confirm the presence of several cell line specific D-loop mtSNVs, as a means of confirming mitochondrial transplantation between cells, but complete D-loop or whole mtDNA sequencing data was not reported^80^*.* Whilst this does limit out ability to compare the relative application of two emerging subcellular isolation technologies, both have demonstrated the ability to accurately isolate subcelluar mitochondria for mtDNA sequencing. Whilst nanobiospy is currently the first technology to be demonstrated for subcellular sequencing of mtDNA acquired from culture cells^39^ and tissue, it is likely that an array of subcellular isolation technologies will faciliate a transition towards subcellular omics.

Having validated the nanobiopsy technique to investigate subcellular heteroplasmy from distinct foci, an obvious next step is to investigate localised patterns in subcellular heteroplasmy within disease tissues to elucidate specific mechanisms responsible for clonal expansion of mtDNA mutations^18,29^. Our approach allows the investigation of subcellular heteroplasmy in other tissues and cultured cells as a means of investigating spatial and temporal patterns in mtDNA mutation distribution. Recent advancements in Oxford Nanopore Sequencing technologies also offer the future potential for enrichment free ‘pore-to-pore’ sequencing^88^. Nanobiopsy in combination with existing and emerging sequencing technologies will allow us to harness the untapped potential of these platforms to perform subcellular sequencing^84^, this is increasingly vital as we move away from the era of single-cell omics towards subcellular-omics^89^.

### Supplementary Information

Supplementary Information.

## Data Availability

All datasets used and/or analysed in this study are available from the corresponding author(s) on request.
