# Supplementary material for: Nanobiopsy investigation of the subcellular mtDNA heteroplasmy in human tissues
Source: Sci Rep. 2024 Jun 14;14:13789. doi: 10.1038/s41598-024-64455-0 (PMC11178779; doi:10.1038/s41598-024-64455-0)
Supplement: Supplementary file 1 — Supplementary Information. [file 41598_2024_64455_MOESM1_ESM.docx]

*Nanobiopsy investigation of the subcellular mtDNA heteroplasmy in human tissues.*

Dr Alexander Bury^1, 3, 4, 5^, Dr Angela Pyle^2^, Dr Amy E. Vincent^2,3*^, Dr Paolo Actis^4,5*^, Prof. Gavin Hudson^1,3*^.

^1^Wellcome Centre for Mitochondrial Research, Biosciences Institute, Faculty of Medical Sciences, Newcastle University, Newcastle, United Kingdom

^2^Wellcome Centre for Mitochondrial Research, Translational and Clinical Research Institute, Faculty of Medical Sciences, Newcastle University, Newcastle, United Kingdom.

^3^NIHR Biomedical Research Centre, Faculty of Medical Science, Newcastle University, United Kingdom.

^4^ School of Electronic and Electrical Engineering and Pollard Institute, University of Leeds, Leeds, United Kingdom.

^5^Bragg Centre for Materials Research, Leeds, United Kingdom.

^*^Corresponding authors.

**Supplementary information**

**Table S1. Subcellular and single-cell biopsies.** Summary of biopsies taken from muscle tissue obtained from a healthy patient taken during elective ACL surgery. The table highlights the muscle fibre and the corresponding number of biopsies that were taken. The proportion of biopsies successfully enriched using PCR is also shown as well as the subcellular foci from which the biopsy was samples from (nanobiopsy samples only). Biopsies that were successfully enriched using PCR are highlighted in **bold**. PN = perinuclear foci, IMF = intermyofibrillar, SS – subsarcolemmal.

| **Fibre** | **Biopsies**  **Successfully isolated** | **N^o^ Biopsies**  **Successfully enriched (PCR)** | **Foci** |
| --- | --- | --- | --- |
| Subcellular biopsy (nanobiopsy) | | | |
| **1** | 2 | 3 (1/4, 25%) | PN |
|  | **3** |  | **PN** |
|  | 4 |  | IMF |
|  | 5 |  | SS |
| **2** | 10 | 13 (1/4, 25%) | PN |
|  | 11 |  | IMF |
|  | 12 |  | IMF |
|  | **13** |  | SS |
| **3** | **14** | 14, 17-19 (4/6, 66.6%) | IMF |
|  | 15 |  | **SS** |
|  | 16 |  | SS |
|  | **17** |  | **IMF** |
|  | **18** |  | **IMF** |
|  | **19** |  | **PN** |
| **4** | **20** | 20, 21 (2/4, 50%) | **IMF** |
|  | **21** |  | **IMF** |
|  | 22 |  | PN |
|  | 23 |  | SS |
| **5** | 24 | 25, 26 (2/4, 50%) | PN |
|  | **25** |  | **SS** |
|  | **26** |  | **SS** |
|  | 27 |  | IMF |
| **6** | 48 | 51 (1/4, 25%) | IMF |
|  | 49 |  | PN |
|  | 50 |  | SS |
|  | **51** |  | **IMF** |
| 7 | 6 | (0, 0%) | SS |
|  | 7 |  | IMF |
|  | 8 |  | PN |
|  | 9 |  | PN |
| 8 | 28 | (0, 0%) | PN |
|  | 29 |  | SS |
|  | 30 |  | IMF |
|  | 31 |  | SS |
| 9 | 32 | (0, 0%) | PN |
|  | 33 |  | IMF |
|  | 34 |  | SS |
|  | 35 |  | SS |
| 10 | 36 | (0, 0%) | SS |
|  | 37 |  | PN |
|  | 38 |  | IMF |
|  | 39 |  | IMF |
| 11 | 40 | (0, 0%) | PN |
|  | 41 |  | SS |
|  | 42 |  | IMF |
|  | 43 |  | SS |
| 12 | 44 | (0, 0%) | IMF |
|  | 45 |  | SS |
|  | 46 |  | PN |
|  | 47 |  | PN |
| **Total** | ***n* = 50** | **11/50, 22%** | - |
| Single-cell biopsy (LCM) | | | |
| 13 | 1 | 0/1, 0% | - |
| 14 | 2 | 0/1, 0% | - |
| 15 | 3 | 0/1, 0% | - |
| 16 | 4 | 0/1, 0% | - |
| 17 | 5 | 0/1, 0% | - |
| 18 | 6 | 0/1, 0% | - |
| 19 | 7 | 0/1, 0% | - |
| 20 | 8 | 0/1, 0% | - |
| 21 | 9 | 0/1, 0% | - |
| 22 | 10 | 0/1, 0% | - |
| 23 | 11 | 0/1, 0% | - |
| 24 | 12 | 0/1, 0% | - |
| 25 | 13 | 0/1, 0% | - |
| 26 | 14 | 0/1, 0% | - |
| 27 | 15 | 0/1, 0% | - |
| 28 | 16 | 0/1, 0% | - |
| 29 | 17 | 0/1, 0% | - |
| 30 | 18 | 0/1, 0% | - |
| **31** | **19** | 1/1, 100% | - |
| **32** | **20** | 1/1, 100% | - |
| **33** | **21** | 1/1, 100% | - |
| **34** | **22** | 1/1, 100% | - |
| **Total** | ***n* = 22** | **4/22, 18%** | - |


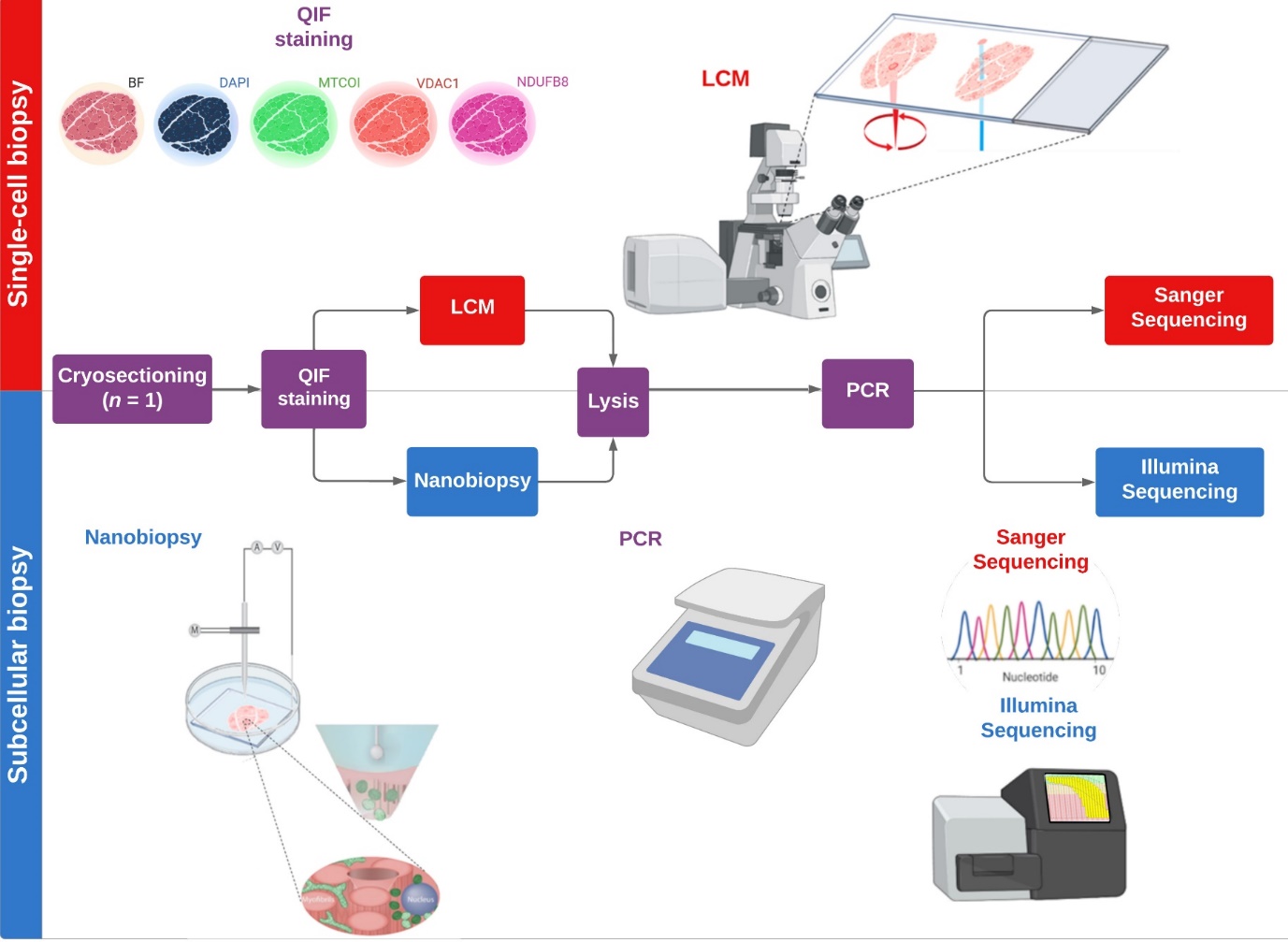


**Figure S1. Workflow for subcellular and single-cell biopsies.** All biopsies were obtained from tissue sections taken from a single healthy patient. Sections then underwent quadimmunolfourescent (QIF) staining followed by acquisutiion of single-cell or subcellular biopsies through laser capture-microdissection (LCM) or nanobiospy, respectively. Biopsies were lysed and mtDNA was enirched using targeted PCR of the mtDNA control region. Following mtDNA purification and library preparation, mtDNA from singe-cell lysate was Sanger sequenced whilst nanobiopsied mtDNA underwent Illumina sequencing.
